# Zero-Shot Neural Decoding with Semi-Supervised Multi-View Embedding

**DOI:** 10.3390/s23156903

**Published:** 2023-08-03

**Authors:** Yusuke Akamatsu, Keisuke Maeda, Takahiro Ogawa, Miki Haseyama

**Affiliations:** 1 Graduate School of Information Science and Technology, Hokkaido University, N-14, W-9, Kita-ku, Sapporo 060-0814, Hokkaido, Japan; 2 Faculty of Information Science and Technology, Hokkaido University, N-14, W-9, Kita-ku, Sapporo 060-0814, Hokkaido, Japan

**Keywords:** neural decoding, functional magnetic resonance imaging (fMRI), zero-shot learning, multi-view learning, semi-supervised learning, Bayesian inference, generative model, probabilistic model

## Abstract

Zero-shot neural decoding aims to decode image categories, which were not previously trained, from functional magnetic resonance imaging (fMRI) activity evoked when a person views images. However, having insufficient training data due to the difficulty in collecting fMRI data causes poor generalization capability. Thus, models suffer from the projection domain shift problem when novel target categories are decoded. In this paper, we propose a zero-shot neural decoding approach with semi-supervised multi-view embedding. We introduce the semi-supervised approach that utilizes additional images related to the target categories without fMRI activity patterns. Furthermore, we project fMRI activity patterns into a multi-view embedding space, i.e., visual and semantic feature spaces of viewed images to effectively exploit the complementary information. We define several source and target groups whose image categories are very different and verify the zero-shot neural decoding performance. The experimental results demonstrate that the proposed approach rectifies the projection domain shift problem and outperforms existing methods.

## 1. Introduction

Neural decoding has enabled the interpretation of a person’s cognitive state from evoked brain activity. This attempt makes a significant contribution to the development of brain–computer interfaces (BCIs) [1] that would establish communication between computer systems and human brain activity. Several machine learning methods [2,3,4] have revealed what a person viewed from evoked brain activity. In these methods, functional magnetic resonance imaging (fMRI) activity patterns that were measured when a subject viewed several images (e.g., fish, chair, and face) were classified into a valid image category. Since these methods focused on the relationship between fMRI activity patterns and image categories used in the training phase, the predicted categories were restricted to trained categories only. It is not feasible to acquire fMRI activity patterns for all possible categories; therefore, the results of these methods are significantly limited.

**Figure 1:**
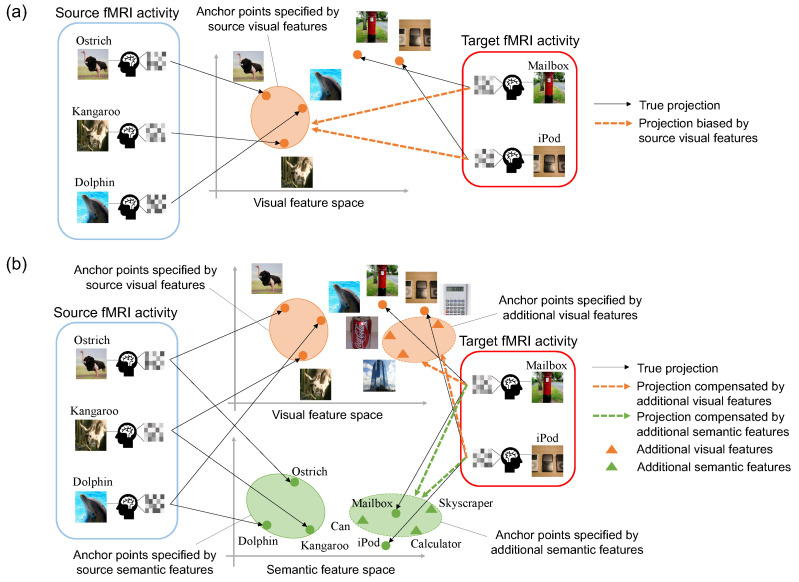
(**a**) The projection domain shift problem in zero-shot neural decoding. When the source categories (i.e., ostrich, kangaroo, and dolphin) and the target categories (i.e., mailbox and iPod) are potentially different, the projections that connect fMRI activity with the visual feature space are biased towards the source categories. This bias could keep target fMRI activity embeddings away from the actual target category embeddings. (**b**) Our proposed semi-supervised multi-view embedding. We embedded additional visual and semantic features extracted from images related to the target categories without fMRI activity patterns. By introducing additional visual and semantic features, the biased projections towards the source categories are compensated, and the projection domain shift problem is alleviated.

To overcome this limitation, zero-shot neural decoding approaches attempt to decode viewed images [5,6,7,8,9,10] and meanings of presented words [11,12,13] that were not trained previously from corresponding brain activity. For example, the studies [6,7,8,14] estimated mappings between fMRI activity patterns and visual features that were extracted from the viewed images via convolutional neural networks (CNN) [15]. Then, visual features were predicted from fMRI activity measured when a subject viewed a novel category. Finally, the decoding was made feasible by comparing the predicted features with visual features from various categories. On the other hand, zero-shot learning, which aims to recognize novel classes without labeled training samples, suffers from the projection domain shift problem [16,17,18]. Zero-shot learning can be considered as transfer learning that transfers the knowledge from the training/source domain to the test/target domain. When two domains are potentially unrelated, the mappings learned from one source domain may not correctly capture the relationship of the target domain, and this is known as the projection domain shift problem (see Figure 1a). Furthermore, collecting brain activity patterns is a very laborious task since the measurement requires a heavy burden on subjects. Thus, the number of source categories is small, and source categories may be limited to certain categories only. Therefore, the projection domain shift problem is remarkable, resulting in poor decoding performance.

In this paper, we propose a zero-shot neural decoding approach with semi-supervised multi-view embedding. To rectify the projection domain shift problem, we introduce a semi-supervised framework that utilizes images related to the target categories without fMRI activity patterns. Our framework can be seen as domain adaptation relying on images from the target domain, which can be collected at a reasonable cost. Furthermore, we project fMRI activity patterns into a multi-view embedding space: the visual feature space and the semantic feature space. Specifically, visual features are extracted from images via CNN, and semantic features are extracted from image categories via a distributed word representation model. Our semi-supervised multi-view approach can consider different feature spaces that contain complementary information while introducing additional visual and semantic features extracted from images related to the target categories (see Figure 1b). Most importantly, fMRI activity patterns corresponding to additional visual and semantic features are not necessary for the proposed method.

**Figure 2:**
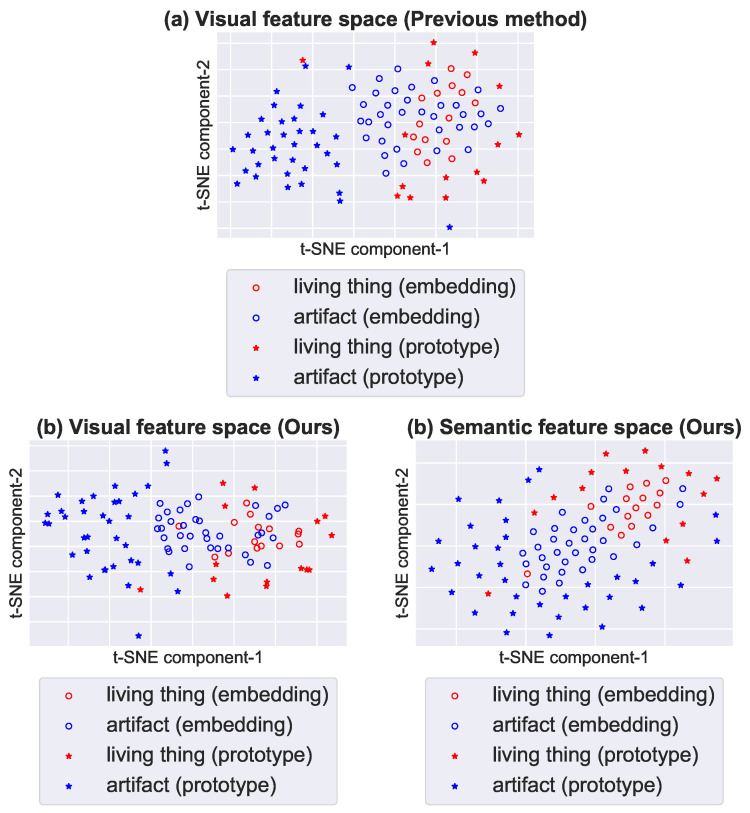
(**a**) The visual feature space by the previous method [6] and (**b**) the visual and semantic feature spaces by the proposed model. As seen in Figure 1, source categories belong to only *living thing*, and target categories of *living thing* (red circles) and *artifact* (blue circles) are projected into the embedding space. Prototypes of *living thing* (red stars) and *artifact* (blue stars) represent the ground truth embeddings.

Towards the realization of our approach, we construct a semi-supervised multi-view generative model. Our proposed model assumes that fMRI activity, visual features, and semantic features are generated from a shared latent variable under a Bayesian framework [19,20]. Furthermore, additional visual and semantic features are incorporated into the model to solve the projection domain shift problem. We consider unobserved fMRI activity patterns corresponding to additional features as missing values and estimate these missing values while optimizing model parameters. Figure 2 demonstrates the embedding space in the previous method [6] and the proposed model by plotting visual and semantic features predicted from fMRI activity (we used fMRI activity collected from subject 3 in the publicly available fMRI dataset [6]) onto two dimensions using t-SNE [21]. In the visual feature space of the method [6] illustrated in Figure 2a, the target category embeddings of *artifact* are biased towards the source categories of *living thing* and are separated from their prototypes. On the other hand, this bias is alleviated in the visual feature space of our method, and target category embeddings of *artifact* are projected properly in the semantic feature space illustrated in Figure 2b. We solve the projection domain shift problem by the semi-supervised approach and consider a better feature space by the multi-view embedding approach.

The main contributions of this paper are as follows:

- To the best of our knowledge, this is the first study to address the projection domain shift problem in zero-shot neural decoding. For this problem, we introduce the semi-supervised framework that employs images related to the target categories without fMRI activity patterns.
- We propose multi-view embeddings that associate fMRI activity patterns with visual features from images and semantic features from image categories.
- We address the difficulty in collecting fMRI data and estimate unobserved fMRI activity patterns in a fully probabilistic manner [22]. Furthermore, the Bayesian framework of our model automatically selects a small set of appropriate components from high dimensional fMRI voxels.

## 2. Related Work

### 2.1. Multi-View Learning

Multi-view learning [23,24] is machine learning which considers learning with multiple views to improve the generalization performance and provide complementary information. Canonical correlation analysis (CCA) [25] is a classical but still powerful tool for analyzing multi-view paired samples. CCA finds projection matrices such that the correlation between projected paired samples in the shared latent space is maximized. Semi-supervised CCA (SemiCCA) [26] utilizes additional unpaired samples to prevent the performance degradation of CCA when the number of paired samples is limited. The CCA framework is also applied to neural decoding [7,14], where pairs of fMRI data and visual features of images that a subject viewed are associated. The method in [14] predicts visual features from fMRI data utilizing CCA to estimate the viewed image categories. The method in [7] introduces semi-supervised fuzzy discriminative CCA (Semi-FDCCA), where additional images not used in measuring fMRI data are utilized by SemiCCA and the similarity information of image categories is incorporated. However, one of the central problems in CCA is the model selection or how to select the dimensions to be retained [27]. In particular, since the number of dimensions of fMRI data is high, dimension reduction must be applied before input to the CCA, resulting in performance degradation.

The proposed model is constructed under the same framework as Bayesian canonical correlation analysis (BCCA) [19,27] and group factor analysis (GFA) [20]. BCCA is a generative model that links two observed variables via a shared latent variable, and GFA also treats more than two observed variables. BCCA and GFA introduce automatic relevance determination (ARD) [28] via the Bayesian approach, which automatically selects the appropriate dimensions from the data. Therefore, we can automatically select a small set of appropriate components from high dimensional fMRI voxels. The proposed model handles three observed variables (i.e., fMRI activity, visual features, and semantic features) and can be easily extended to semi-supervised learning. We also refer to the proposed model without the semi-supervised learning scenario as the multi-view generative model (MGM) [29].

### 2.2. Zero-Shot Learning

Zero-shot learning (ZSL) is the recognition of novel visual categories without labeled training samples for visual recognition. Inspired by humans’ ability to recognize a new object category without ever seeing a visual instance, ZSL aims to recognize a visual instance of a new category that has never been seen before [17]. Common ZSL methods learn a projection function from a visual feature space of images to a semantic embedding space (e.g., a semantic attribute space or a semantic word vector space) using the labeled training data consisting of seen classes only. At test time for recognizing unseen objects, this projection function is then used to project the visual representation of an unseen class image into the semantic embedding space. However, ZSL models mostly suffer from the projection domain shift problem [16]. That is, if the projection is learned only from the seen classes, the projections of unseen class images are likely to be misplaced (shifted) due to the bias of the seen classes [18]. Zero-shot neural decoding also suffers from the domain shift problem when projecting fMRI activity to the visual feature space and semantic feature space.

### 2.3. Neural Decoding

Neural decoding (or brain decoding) is the task of estimating images or words that a person sees or imagines from his/her brain activity (especially fMRI activity). In the traditional neural decoding methods [2,3,4], fMRI activity that was measured when a person viewed an image was classified into an image category, which enables the estimation of the viewed object. Subsequent neural decoding methods [5,6,7,8,9,10,11,12,13] estimate images or words that were not trained before, i.e., zero-shot neural decoding, by projecting fMRI activity into an intermediate-level feature space (e.g., visual feature space [6] and semantic feature space [12,13]). Recent neural decoding approaches [30,31,32,33] employ semi-supervised learning to utilize additional images without corresponding fMRI activity patterns. Beliy et al. [30] aimed to reconstruct high-quality images from evoked fMRI activity while viewing images. Their method utilizes unlabeled data (i.e., images without fMRI recording, and fMRI recording without images) to train fMRI-to-image reconstruction networks. Akamatsu et al. [31] and Du et al. [32] aimed to accurately estimate viewed image categories from evoked fMRI activity. They associate fMRI activity with visual features and semantic [31] or textual features [32] extracted from image categories. They also introduce semi-supervised learning by incorporating additional visual and semantic or textual features without fMRI activity. Liu et al. [33] proposed BrainCLIP, which unifies the visual stimulus classification and image reconstruction from fMRI activity. They leverage the semantic space of CLIP [34] learned from a large-scale vision–language corpus to perform neural decoding tasks. The method is pre-trained with large-scale unlabeled images without fMRI activity.

This paper is an extended version of the previous work [31]. This work aims to address the projection domain shift problem in zero-shot neural decoding, especially when the source domain and the target domain are potentially unrelated. Specifically, we define several source and target groups (e.g., mammal, device, and structure) and validate their decoding performances in zero-shot learning settings. In the previous works [31,32,33], image categories related to the target group were included in the source group (e.g., source: dolphin, airship, and beer mug; target: killer whale, airliner, and coffee mug, respectively). On the other hand, the target and source groups are very different in our setting; therefore, we are tackling a more challenging problem than in previous works.

## 3. Proposed Method

Our proposed method consists of the following three phases: the construction of semi-supervised multi-view generative model, the optimization of the model parameters, and the decoding of viewed image categories from evoked fMRI activity.

### 3.1. Semi-Supervised Multi-View Generative Model

**Figure 3:**
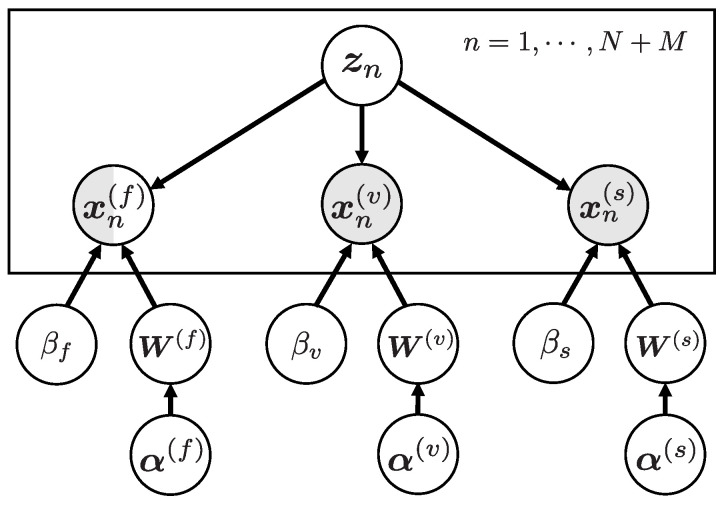
Graphical model of the proposed model. The gray nodes represent observed variables and the white nodes represent model parameters. The mixture node of gray and white includes both observed variables and model parameters (i.e., missing values).

Suppose that $X^{\left(f\right)}=\left[x_{1}^{\left(f\right)},\cdots ,x_{N}^{\left(f\right)}\right]\in R^{D_{f}\times N}$, $X^{\left(v\right)}=\left[x_{1}^{\left(v\right)},\cdots ,x_{N}^{\left(v\right)}\right]\in R^{D_{v}\times N}$, and $X^{\left(s\right)}=\left[x_{1}^{\left(s\right)},\cdots ,x_{N}^{\left(s\right)}\right]\in R^{D_{s}\times N}$ represent fMRI activity patterns, visual features, and semantic features, respectively. Here, $D_{f}$, $D_{v}$, and $D_{s}$ denote the dimensions of each sample of $X^{\left(f\right)}$, $X^{\left(v\right)}$, and $X^{\left(s\right)}$, respectively. *N* denotes the size of training samples. Visual features $X^{\left(v\right)}$ are extracted from viewed images via VGG19 [35], which is a CNN model that was used in a previous neural decoding study [36]. Semantic features $X^{\left(s\right)}$ are extracted from viewed image categories based on a word2vec model [37]. Furthermore, suppose that $X_{\mathrm{add}}^{\left(v\right)}=\left[x_{N+1}^{\left(v\right)},\cdots ,x_{N+M}^{\left(v\right)}\right]\in R^{D_{v}\times M}$ and $X_{\mathrm{add}}^{\left(s\right)}=\left[x_{N+1}^{\left(s\right)},\cdots ,x_{N+M}^{\left(s\right)}\right]\in R^{D_{s}\times M}$ represent additional visual and semantic features from images related to the target categories, respectively. Here, a set of additional visual and semantic features is obtained from each category, i.e., *M* denotes the number of additional image categories. Our approach considers unobserved fMRI activity patterns corresponding to additional visual and semantic features as missing values. These missing values $X_{\mathrm{miss}}^{\left(f\right)}=\left[x_{N+1}^{\left(f\right)},\cdots ,x_{N+M}^{\left(f\right)}\right]\in R^{D_{f}\times M}$ are estimated by Bayesian inference [22] while we are optimizing the model parameters. Hereafter, the observed and missing variables are redefined as ${\hat{X}}^{\left(f\right)}=\left[X^{\left(f\right)},X_{\mathrm{miss}}^{\left(f\right)}\right]\in R^{D_{f}\times \left(N+M\right)}$, ${\hat{X}}^{\left(v\right)}=\left[X^{\left(v\right)},X_{\mathrm{add}}^{\left(v\right)}\right]\in R^{D_{v}\times \left(N+M\right)}$, and ${\hat{X}}^{\left(s\right)}=\left[X^{\left(s\right)},X_{\mathrm{add}}^{\left(s\right)}\right]\in R^{D_{s}\times \left(N+M\right)}$. Note that [·,·] represents the horizontal concatenation. We also introduce latent variables $Z=\left[z_{1},\cdots ,z_{N+M}\right]\in R^{D_{z}\times \left(N+M\right)}$ that generate fMRI activity ${\hat{X}}^{\left(f\right)}$, visual features ${\hat{X}}^{\left(v\right)}$, and semantic features ${\hat{X}}^{\left(s\right)}$, where $D_{z}$ is the dimension of each sample of Z. In our model, $D_{z}$ was set to the smallest dimension of ${\hat{X}}^{\left(f\right)}$, ${\hat{X}}^{\left(v\right)}$, and ${\hat{X}}^{\left(s\right)}$. The prior distributions of the missing values $X_{\mathrm{miss}}^{\left(f\right)}$ and latent variables Z are initialized by random variables following a multivariate normal distribution as

(1) $$\begin{array}{cc} & \;p_{0}\left(X_{\mathrm{miss}}^{\left(f\right)}\right)=\prod_{n=N+1}^{N+M}N\left(x_{n}^{\left(f\right)}|0,I_{D_{f}}\right), \end{array}$$

(2) $$\begin{array}{cc} & p_{0}\left(Z\right)=\prod_{n=1}^{N+M}N\left(z_{n}|0,I_{D_{z}}\right), \end{array}$$

where N(x|μ,Σ) represents a multivariate Gaussian distribution with a mean μ and a covariance matrix Σ. 0 denotes the zero vector and $I_{D}$ denotes the identity matrix with D×D size. Our proposed model assumes that fMRI activity ${\hat{X}}^{\left(f\right)}$, visual features ${\hat{X}}^{\left(v\right)}$, and semantic features ${\hat{X}}^{\left(s\right)}$ are generated from shared latent variables Z by the following likelihood function:

(3) $$\begin{array}{cc} p({\hat{X}}^{\left(k\right)}|W^{\left(k\right)},Z) & =\prod_{n=1}^{N+M}N\left(x_{n}^{\left(k\right)}|W^{\left(k\right)}z_{n},\beta_{k}^{-1}I_{D_{k}}\right), \end{array}$$

where k∈{f,v,s}. In Equation (3), $W^{\left(k\right)}\in R^{D_{k}\times D_{z}}$ is a projection matrix that connects Z with ${\hat{X}}^{\left(k\right)}$, and $\beta_{k}^{-1}$ is the scalar variable representing noise variance of ${\hat{X}}^{\left(k\right)}$. The prior distribution of the projection matrix $W^{\left(k\right)}$ and its hyperprior distribution $\alpha^{\left(k\right)}$ are assumed as

(4) $$\begin{array}{cc} & \;p_{0}\left(W^{\left(k\right)}|\alpha^{\left(k\right)}\right)=\prod_{i=1}^{D_{k}}\prod_{j=1}^{D_{z}}N\left(W_{i,j}^{\left(k\right)}|0,{\alpha_{i,j}^{\left(k\right)}}^{-1}\right), \end{array}$$

(5) $$\begin{array}{cc} & p_{0}\left(\alpha^{\left(k\right)}\right)=\prod_{i=1}^{D_{k}}\prod_{j=1}^{D_{z}}G\left(\alpha_{i,j}^{\left(k\right)}|{\bar{\alpha }}_{0(i,j)}^{\left(k\right)},\gamma_{0(i,j)}^{\left(k\right)}\right), \end{array}$$

where ${\alpha_{i,j}^{\left(k\right)}}^{-1}$ is a variance of the elements in $W^{\left(k\right)}$. In Equation (5), we introduce hyperprior distribution for the inverse variances $\alpha^{\left(k\right)}$, and $G(\cdot |\bar{\alpha },\gamma )$ represents the Gamma distribution with a mean $\bar{\alpha }$ and a shape parameter γ. In our model, all of the means ${\bar{\alpha }}_{0(i,j)}^{\left(k\right)}$ and the shape parameters $\gamma_{0(i,j)}^{\left(k\right)}$ were set to 1 and 0, respectively, as in the previous study [19]. This form of prior and hyperprior distributions is motivated by the idea of automatic relevance determination (ARD). Since ARD automatically selects important components in the model, we can extract a small number of appropriate features from observed variables. Especially, this sparse representation is effective for fMRI data analysis since fMRI data have thousands of voxels and it is reported that only a small number of voxels is relevant to a visual stimulus [19,38]. This ARD form avoids overfitting due to the high dimensionality and improves generalization performance. Finally, the prior distribution of inverse variance $\beta_{k}$ is assumed to be an uninformative prior described as $p_{0}\left(\beta_{k}\right)=\beta_{k}^{-1}$. The graphical model of the proposed model is illustrated in Figure 3. The meaning and role of the observed variables and model parameters are summarized in Table 1.

### 3.2. Optimization of Model Parameters via Variational Inference

In this subsection, we estimate the missing values $X_{\mathrm{miss}}^{\left(f\right)}$ and optimize model parameters Z, $W^{\left(k\right)}$, $\alpha^{\left(k\right)}$, and $\beta_{k}$(k∈{f,v,s}). Hereafter, the observed variables (except for the missing values), the projection matrices, and the inverse variances are summarized as $X=\{X^{\left(f\right)},{\hat{X}}^{\left(v\right)},{\hat{X}}^{\left(s\right)}\}$, $W=\{W^{\left(f\right)},W^{\left(v\right)},W^{\left(s\right)}\}$, and $\Theta =\{\alpha^{\left(f\right)},\alpha^{\left(v\right)},\alpha^{\left(s\right)},\beta_{f},\beta_{v},\beta_{s}\}$, respectively. We optimize $X_{\mathrm{miss}}^{\left(f\right)}$, Z, W, and Θ by calculating a posterior distribution $p(X_{\mathrm{miss}}^{\left(f\right)},Z,W,\Theta |X)$. Since this posterior distribution cannot be calculated analytically, we approximate it by using variational inference [39]. In variational inference, we consider an approximate distribution $q(X_{\mathrm{miss}}^{\left(f\right)},Z,W,\Theta )$ as a factorized form described as $q\left(X_{\mathrm{miss}}^{\left(f\right)},Z,W,\Theta \right)\approx q\left(X_{\mathrm{miss}}^{\left(f\right)}\right)q\left(Z\right)q\left(W\right)q\left(\Theta \right)$. Then the approximate distribution is optimized so that the Kullback–Leibler (KL) divergence $D_{KL}\left(q,p\right)$ between the approximate distribution and the true posterior distribution can be minimized. This optimization is equivalent to the maximization of a lower bound L(q) as follows:

(6) $$\begin{array}{cc} L(q) & =\log p\left(X\right)-D_{KL}\left(q,p\right)\\ & =\int q\left(X_{\mathrm{miss}}^{\left(f\right)}\right)q\left(Z\right)q\left(W\right)q\left(\Theta \right)\log\frac{p(X,X_{\mathrm{miss}}^{\left(f\right)},Z,W,\Theta )}{q\left(X_{\mathrm{miss}}^{\left(f\right)}\right)q\left(Z\right)q\left(W\right)q\left(\Theta \right)}dX_{\mathrm{miss}}^{\left(f\right)}dZdWd\Theta . \end{array}$$

The distributions of $q(X_{\mathrm{miss}}^{\left(f\right)})$, q(Z), $q(W^{\left(k\right)})$, $q(\alpha^{\left(k\right)})$, and $q(\beta_{k})$(k∈{f,v,s}) are updated iteratively to maximize the lower bound L(q).

First, we update the distribution of the projection matrix $q(W^{\left(k\right)})$ according to variational inference as

(7) $$\begin{array}{c} q\left(W^{\left(k\right)}\right)=\prod_{i=1}^{D_{k}}\prod_{j=1}^{D_{z}}N\left(W_{i,j}^{\left(k\right)}|{\bar{W}}_{i,j}^{\left(k\right)},{\sigma_{i,j}^{\left(k\right)}}^{-1}\right),\;k\in \left\{f,v,s\right\} \end{array}$$

where

(8) $$\begin{array}{cc} & \;\sigma_{i,j}^{\left(k\right)}={\bar{\beta }}_{k}\left(\sum_{n=1}^{N+M}{\bar{z}}_{n(j)}^{2}+\left(N+M\right)\sigma_{z(j,j)}^{-1}\right)+{\bar{\alpha }}_{i,j}^{\left(k\right)}, \end{array}$$

(9) $$\begin{array}{cc} & {\bar{W}}_{i,j}^{\left(k\right)}={\bar{\beta }}_{k}{\sigma_{i,j}^{\left(k\right)}}^{-1}\sum_{n=1}^{N+M}x_{n(i)}^{\left(k\right)}{\bar{z}}_{n(j)}. \end{array}$$

In Equations (8) and (9), ${\bar{z}}_{n(j)}$ represents the mean of the *j*th variable of $z_{n}$. Next, the distribution of the latent variables q(Z) is updated as

(10) $$\begin{array}{c} q\left(Z\right)=\prod_{n=1}^{N+M}N\left(z_{n}|{\bar{z}}_{n},\sigma_{z}^{-1}\right), \end{array}$$

where

(11) $$\begin{array}{cc} & \;\sigma_{z}={\bar{\beta }}_{f}\left({{\bar{W}}^{\left(f\right)}}^{T}{\bar{W}}^{\left(f\right)}+\sigma_{W^{\left(f\right)}}^{-1}\right)+\\ & \;\;\;\;\;\;\;{\bar{\beta }}_{v}\left({{\bar{W}}^{\left(v\right)}}^{T}{\bar{W}}^{\left(v\right)}+\sigma_{W^{\left(v\right)}}^{-1}\right)+{\bar{\beta }}_{s}\left({{\bar{W}}^{\left(s\right)}}^{T}{\bar{W}}^{\left(s\right)}+\sigma_{W^{\left(s\right)}}^{-1}\right)+I_{D_{z}}, \end{array}$$

(12) $$\begin{array}{cc} & {\bar{z}}_{n}=\sigma_{z}^{-1}\left({\bar{\beta }}_{f}{{\bar{W}}^{\left(f\right)}}^{T}x_{n}^{\left(f\right)}+{\bar{\beta }}_{v}{{\bar{W}}^{\left(v\right)}}^{T}x_{n}^{\left(v\right)}+{\bar{\beta }}_{s}{{\bar{W}}^{\left(s\right)}}^{T}x_{n}^{\left(s\right)}\right). \end{array}$$

In Equation (11), $\sigma_{W^{\left(k\right)}}(k\in \left\{f,v,s\right\}$) is defined as

(13) $$\begin{array}{c} \sigma_{W^{\left(k\right)}}=\mathrm{diag}\left(\sum_{i=1}^{D_{k}}\sigma_{i,1}^{\left(k\right)},\cdots ,\sum_{i=1}^{D_{k}}\sigma_{i,D_{z}}^{\left(k\right)}\right). \end{array}$$

Furthermore, the distribution of the missing values $q(X_{\mathrm{miss}}^{\left(f\right)})$ is updated as

(14) $$\begin{array}{c} q\left(X_{\mathrm{miss}}^{\left(f\right)}\right)=\prod_{n=N+1}^{N+M}N\left(x_{n}|{\bar{W}}^{\left(f\right)}{\bar{z}}_{n},\beta_{f}^{-1}I_{D_{f}}\right). \end{array}$$

In Equation (14), we estimate the missing values, i.e., unobserved fMRI activity patterns, by using the current model parameters $W^{\left(f\right)}$, Z, and $\beta_{f}$. Finally, we update the distribution of the variances $q(\alpha^{\left(k\right)})$ and $q(\beta_{k})$(k∈{f,v,s}). The distribution $q(\alpha^{\left(k\right)})$ is updated as

(15) $$\begin{array}{cc} q(\alpha^{\left(k\right)}) & =\prod_{i=1}^{D_{k}}\prod_{j=1}^{D_{z}}G\left(\alpha_{i,j}^{\left(k\right)}|{\bar{\alpha }}_{\left(i,j\right)}^{\left(k\right)},\gamma_{\left(i,j\right)}^{\left(k\right)}\right),\;k\in \left\{f,v,s\right\} \end{array}$$

where

(16) $$\begin{array}{cc} & \;\gamma_{\left(i,j\right)}^{\left(k\right)}=\frac{1}{2}+\gamma_{0(i,j)}^{\left(k\right)}, \end{array}$$

(17) $$\begin{array}{cc} & {{\bar{\alpha }}_{i,j}^{\left(k\right)}}^{-1}={\gamma_{\left(i,j\right)}^{\left(k\right)}}^{-1}\left(\frac{1}{2}{{\bar{W}}_{i,j}^{\left(k\right)}}^{2}+\frac{1}{2}{\sigma_{i,j}^{\left(k\right)}}^{-1}+\gamma_{0(i,j)}^{\left(k\right)}\sigma_{0(i,j)}^{(k)-1}\right). \end{array}$$

In our model, $\sigma_{0(i,j)}^{(k)-1}$ was set to 0 as in [19]. The distribution $q(\beta_{k})$ is updated as

(18) $$\begin{array}{cc} q(\beta_{k}) & =G\left(\beta_{k}|{\bar{\beta }}_{k},\gamma_{\beta_{k}}\right),\;k\in \left\{f,v,s\right\} \end{array}$$

where

(19) $$\begin{array}{cc} & \;\gamma_{\beta_{k}}=\frac{1}{2}D_{k}\left(N+M\right), \end{array}$$

(20) $$\begin{array}{cc} & \;{\bar{\beta }}_{k}^{-1}=\frac{1}{2}\gamma_{\beta_{k}}^{-1}\{\sum_{n=1}^{N+M}\left|\right|x_{n}^{\left(k\right)}-{\bar{W}}^{\left(k\right)}{\bar{z}}_{n}{\left|\right|}^{2}+\\ & \;\;\;\;\mathrm{Tr}[\sigma_{W^{\left(k\right)}}^{-1}\left(\sum_{n=1}^{N+M}z_{n}z_{n}^{T}+\left(N+M\right)\sigma_{z}^{-1}\right)+\left(N+M\right)\sigma_{z}^{-1}{{\bar{W}}^{\left(k\right)}}^{T}{\bar{W}}^{\left(k\right)}]\}. \end{array}$$

These update procedures of the model parameters are summarized in Algorithm 1.


**Algorithm 1** Update procedures of model parameters**Input:** Observed variables $X=\{X^{\left(f\right)},{\hat{X}}^{\left(v\right)},{\hat{X}}^{\left(s\right)}\}$
 Initialize $X_{\mathrm{miss}}^{\left(f\right)}$, Z, $W^{\left(k\right)}$, $\alpha^{\left(k\right)}$, and $\beta_{k}$(k∈{f,v,s}) by prior distributions in Equations (1), (2), (4), (5)
 **for** number of training iterations **do**
   Update the projection matrix $W^{\left(k\right)}$ in Equation (7)
   Update the shared latent variables Z in Equation (10)
   Update the missing values $X_{\mathrm{miss}}^{\left(f\right)}$ in Equation (14)
   Update the inverse variances $\alpha^{\left(k\right)}$ in Equation (15)
   Update the inverse variance $\beta_{k}$ in Equation (18)
 **end for**

**Output:** Updated distributions of $X_{\mathrm{miss}}^{\left(f\right)}$, Z, $W^{\left(k\right)}$, $\alpha^{\left(k\right)}$, and $\beta_{k}$(k∈{f,v,s})



### 3.3. Decoding of Viewed Image Categories from fMRI Activity

After the optimization of model parameters, image categories are decoded from fMRI activity evoked by test visual stimuli, i.e., unobserved images. In zero-shot neural decoding settings, the categories of test visual stimuli are not included in the training categories. Suppose that $x_{\mathrm{test}}^{\left(f\right)}\in R^{D_{f}}$ is the fMRI activity measured when a subject viewed a test image. We decode the viewed image category by predicting visual features $x_{\mathrm{test}}^{\left(v\right)}\in R^{D_{v}}$ and semantic features $x_{\mathrm{test}}^{\left(s\right)}\in R^{D_{s}}$ from test fMRI activity $x_{\mathrm{test}}^{\left(f\right)}$. For the prediction of visual and semantic features, we calculate the following predictive distribution constructed from the likelihood function $p(x_{\mathrm{test}}^{\left(i\right)}|W^{\left(i\right)},z_{\mathrm{test}})$, the distribution of projection matrix $q(W^{\left(i\right)})$, and a posterior distribution $p(z_{\mathrm{test}}|x_{\mathrm{test}}^{\left(f\right)})$ as follows:

(21) $$\begin{array}{cc} p\left(x_{\mathrm{test}}^{\left(i\right)}|x_{\mathrm{test}}^{\left(f\right)}\right)=\int & \;{p(x}_{\mathrm{test}}^{\left(i\right)}\left|W^{\left(i\right)},z_{\mathrm{test}}\right)q\left(W^{\left(i\right)}\right)p\left(z_{\mathrm{test}}|x_{\mathrm{test}}^{\left(f\right)}\right)dW^{\left(i\right)}dz_{\mathrm{test}},\;i\in \left\{v,s\right\}. \end{array}$$

In Equation (21), $z_{\mathrm{test}}$ is a latent variable shared by $x_{\mathrm{test}}^{\left(f\right)}$, $x_{\mathrm{test}}^{\left(v\right)}$, and $x_{\mathrm{test}}^{\left(s\right)}$. Since the posterior distribution $p(z_{\mathrm{test}}|x_{\mathrm{test}}^{\left(f\right)})$ is an unknown distribution, we approximate it based on the distribution of the latent variables q(Z) (Equations (10)–(12)). We derive an approximate distribution $\tilde{q}\left(z_{\mathrm{test}}\right)$ by omitting the terms with respect to visual features and semantic features in Equations (10)–(12) as follows:

(22) $$\begin{array}{c} \tilde{q}\left(z_{\mathrm{test}}\right)=N\left(z_{\mathrm{test}}|{\bar{z}}_{\mathrm{test}},\sigma_{z_{\mathrm{test}}}^{-1}\right), \end{array}$$

where

(23) $$\begin{array}{cc} & \;\sigma_{z_{\mathrm{test}}}={\bar{\beta }}_{f}\left({{\bar{W}}^{\left(f\right)}}^{T}{\bar{W}}^{\left(f\right)}+\sigma_{W^{\left(f\right)}}^{-1}\right)+I_{D_{z}}, \end{array}$$

(24) $$\begin{array}{cc} & {\bar{z}}_{\mathrm{test}}={\bar{\beta }}_{f}\sigma_{z_{\mathrm{test}}}^{-1}{{\bar{W}}^{\left(f\right)}}^{T}x_{\mathrm{test}}^{\left(f\right)}. \end{array}$$

Since the multiple integral of Equation (21) cannot be calculated analytically, we replace $q(W^{\left(i\right)})$ with ${\bar{W}}^{\left(i\right)}$ obtained from Equation (9). Moreover, $p(x_{\mathrm{test}}^{\left(i\right)}|W^{\left(i\right)},z_{\mathrm{test}})$ is obtained from the likelihood function in Equation (3). From the above approximations, the predictive distribution in Equation (21) becomes

(25) $$\begin{array}{cc} p(x_{\mathrm{test}}^{\left(i\right)}|x_{\mathrm{test}}^{\left(f\right)}) & \approx N\left(x_{\mathrm{test}}^{\left(i\right)}|{\bar{x}}_{\mathrm{test}}^{\left(i\right)},\sigma_{x_{\mathrm{test}}^{\left(i\right)}}^{-1}\right),\;i\in \left\{v,s\right\}, \end{array}$$

where

(26) $$\begin{array}{cc} & \;\sigma_{x_{\mathrm{test}}^{\left(i\right)}}={\bar{W}}^{\left(i\right)}\sigma_{z_{\mathrm{test}}}^{-1}{{\bar{W}}^{\left(i\right)}}^{T}+{\bar{\beta }}_{i}^{-1}I_{D_{i}}, \end{array}$$

(27) $$\begin{array}{cc} & {\bar{x}}_{\mathrm{test}}^{\left(i\right)}={\bar{\beta }}_{f}{\bar{W}}^{\left(i\right)}\sigma_{z_{\mathrm{test}}}^{-1}{{\bar{W}}^{\left(f\right)}}^{T}x_{\mathrm{test}}^{\left(f\right)}. \end{array}$$

Finally, these predicted visual and semantic features $x_{\mathrm{test}}^{\left(i\right)}$(i∈{v,s}) are obtained by averaging samples ${\bar{x}}_{\mathrm{test}}^{\left(i\right)}$ through many sets of model training and prediction,

(28) $$\begin{array}{c} x_{\mathrm{test}}^{\left(i\right)}=\frac{1}{T}\sum_{t=1}^{T}{\bar{x}}_{\mathrm{test}}^{\left(i\right)},\;i\in \left\{v,s\right\}. \end{array}$$

In our analysis, the number of sets of model training and prediction was set to T=100.

These predicted features are compared with visual and semantic features obtained from various candidate categories. Specifically, we calculate the Pearson correlation coefficient $r^{\left(v\right)}$ between $x_{\mathrm{test}}^{\left(v\right)}$ and visual features obtained from a candidate category as in previous studies [6,8]. We also calculate the Pearson correlation coefficient $r^{\left(s\right)}$ between $x_{\mathrm{test}}^{\left(s\right)}$ and semantic features obtained from a candidate category. $r^{\left(v\right)}$ and $r^{\left(s\right)}$ are calculated separately from visual and semantic features, respectively. The values of $r^{\left(v\right)}$ and $r^{\left(s\right)}$ range from −1 to 1, and higher values indicate that these features are similar. Then these correlation coefficients are integrated as $r^{\left(v+s\right)}=\eta \cdot r^{\left(v\right)}+\left(1-\eta \right)\cdot r^{\left(s\right)}$, where η(0≤η≤1) is the trade-off parameter of balancing visual and semantic features. The decoding of the viewed image category is feasible by identifying a candidate category that scored the max correlation coefficient $r^{\left(v+s\right)}$.

## 4. Experimental Results

### 4.1. Dataset

We utilized a publicly available fMRI dataset [6] in this study. This dataset includes fMRI activity patterns measured when five subjects viewed images collected from ImageNet [40]. In this dataset, fMRI activity patterns that were measured when each subject viewed 1200 images from 150 categories (eight images per category) were collected for the training data. Furthermore, fMRI activity patterns that were measured when each subject viewed 50 images from 50 categories (one image per category), which differ from training categories, were collected for the test data. Since test fMRI activity was measured 35 times for each test image, we used the average fMRI activity patterns across all trials, as in [6]. We used fMRI activity with approximately 4500-dimensional (the number of voxels) vectors from the visual cortex (V1-V4, the lateral occipital complex, fusiform face area, and parahippocampal place area) defined in the study [6]. Visual features with 512-dimensional vectors were extracted from images via the last convolution layer with an average pooling of VGG19 that was pre-trained on ImageNet. Semantic features with 300-dimensional vectors were obtained from image categories via a word2vec that was pre-trained on a Google News dataset. When the categories were not in the word2vec corpus, we utilized hypernyms on ImageNet whose semantic features were available. For example, since the category ‘beer mug’ was not in the corpus, we utilized ‘mug’, which is a hypernym of ‘beer mug’.

### 4.2. Conditions

To validate the effectiveness of the semi-supervised multi-view generative model, we compared the proposed model with the following seven methods: existing neural decoding methods [6,7,8,14], BCCA [19], and the multi-view generative model (MGM). The methods in [6,8] predicted visual features of viewed images from fMRI activity via sparse logistic regression (SLR) [38] and multilayer perceptrons (MLP) [41], respectively. The method in [14] projected fMRI activity and visual features into the same latent space by CCA [25]. The method in [7] constructed semi-supervised fuzzy discriminative CCA (Semi-FDCCA) that incorporated similarities of image categories and visual features from various images into the framework of CCA. BCCA and MGM are in the same framework as our proposed model. BCCA assumes that the number of observed variables is two. Here, we constructed the following two BCCA methods: BCCA-V and BCCA-S. The observed variables of BCCA-V are fMRI activity and visual features, and those of BCCA-S are fMRI activity and semantic features. MGM associated fMRI activity with visual features and semantic features, i.e., the proposed model without a semi-supervised learning scenario. We verified the effectiveness of the multi-view approach by comparing ours with BCCA-V and BCCA-S, and verified the effectiveness of the semi-supervised approach by comparing ours with MGM. In all methods, we standardized fMRI activity, visual features, and semantic features before the training and testing phases. In BCCA-V, BCCA-S, MGM, and the proposed model, the number of training iterations was set to 10. In MGM and the proposed model, the trade-off parameter η was set to the best value from η∈{0,0.1,⋯,1} for each model.

In this experiment, we used identification accuracy to evaluate the decoding performance as in previous studies [6,8]. This evaluation metric represents the accuracy for identifying its ground truth image category between the two candidate categories: one is the ground truth category and another is a randomly selected category (chance level being 50%). This identification was performed based on the correlation coefficient $r^{\left(v+s\right)}$ between predicted features and each candidate category feature. We prepared 10,000 candidate categories (including 50 test categories) randomly selected from ImageNet. Therefore, we performed this identification between all combinations of a ground truth category and one of the other 9,999 candidate categories. Furthermore, we also evaluated by using rank-*n* accuracy to confirm where the correct category is ranked in all candidates. Rank-*n* accuracy refers to the ratio that shows that a correct category is ranked at top-*n* ranks across 10,000 candidates based on the correlation coefficient $r^{\left(v+s\right)}$.

### 4.3. Results and Discussion

We evaluate the decoding performance in several zero-shot neural decoding settings where the source domain and the target domain are potentially unrelated. In this experiment, we classified categories used in the dataset [6] into eight target groups: *mammal, bird, invertebrate, device, container, equipment, structure*, and *commodity*. These groups are defined as subgroups in ImageNet. For zero-shot analysis, several categories that belong to each target group were excluded from the 150 training categories of the dataset [6], and the remaining categories were used for training. Moreover, several categories that belong to each target group were collected from 50 test categories of the dataset [6], and fMRI activity patterns corresponding to these categories were used for test fMRI activity. Furthermore, the proposed model incorporated additional image categories that belong to each target group, which were collected for as many categories as possible from ImageNet. As additional visual features, we used the average visual features extracted from multiple images of an additional category. The numbers of training, test, and additional categories for each target group are described in Table 2. Note that we have eight training samples per training category.

**Decoding performance** Table 3 shows the identification accuracy for each target group. The accuracy was averaged across five subjects. As shown in this table, the proposed model outperforms the seven other methods. By comparing our model with MGM, we confirmed the effectiveness of the semi-supervised approach that employs additional image categories belonging to target groups. We also confirmed that the identification accuracy of SLR and BCCA-V for *mammal* are remarkably low, whereas the accuracy of BCCA-S is comparatively high. These results indicate that *mammal* can be decoded more successfully from the semantic feature space than the visual feature space. On the other hand, the accuracy for other target groups such as *container* and *structure* from the visual feature space is higher than that of the semantic feature space. Therefore, considering a multi-view embedding space is effective since the better feature space depends on each target group. Table 4 presents the rank-*n* (n=100,1000,5000) accuracy in 10,000 random candidate categories. From the table, we also confirmed that the rank accuracy of the proposed model is higher than that of comparative methods and outperforms the chance level.

In Table 3, we used 10,000 candidate categories when identification accuracy was calculated. This evaluation metric indicates how accurately the correct category can be identified from a large number of random categories. Here, we also validate how accurately the correct category can be identified from similar categories (i.e., target groups). Table 5 shows the identification accuracy of comparative methods and the proposed method when the candidate categories are in each target group. For example, when test categories of *mammal* were decoded, we performed identification between all combinations of the ground truth category and one of the candidate categories from *mammal*. As seen in Table 5, the identification accuracy of almost all target groups are degraded in comparison with Table 3, since it is more difficult to identify a ground truth category from similar candidate categories. Nevertheless, the proposed model outperforms comparison methods for all target groups. By comparing with MGM, we confirmed that the utilization of additional visual and semantic features from each target group contributes to the identification from similar candidate categories.

**Figure 4:**
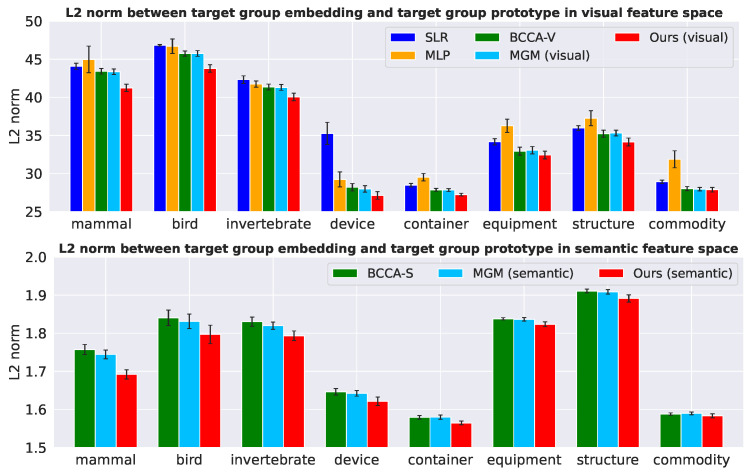
Distance between target group embeddings and their prototypes in the visual and semantic feature spaces. The L2 norm was averaged across five subjects and the error bars represent standard errors.

**Projection domain shift problem** To examine the projection domain shift problem, we visualized the L2 norm between target group embedding and target group prototype in the visual and semantic feature spaces, as seen in Figure 4. Target group embedding is the average vector of embedding features of categories from each target group, and target group prototype is the average vector of ground truth values of categories from each target group. The L2 norm was averaged across five subjects. CCA [14] and Semi-FDCCA [7] were not considered in Figure 4 since these methods calculate the distance between features in the canonical space. From Figure 4, the L2 norm of the proposed model is shorter than that of comparative methods in both the visual and semantic feature spaces. This result shows that the semi-supervised approach reduces the embedding biases towards source categories and realizes better target embeddings towards their prototypes. This alleviation of the projection domain shift problem is effective not only in identifying from a large number of random categories (see Table 3), but also in identifying from similar categories (see Table 5). (Identification accuracy tends to improve when the L2 norm is short. However, the L2 norm is not perfectly consistent with identification accuracy. This is because the L2 norm is calculated purely on the distance of the embedding features, while the identification accuracy is calculated by comparing the embedding features with other candidate features. Since the distribution of candidate features is not uniform but depends on the target group, the relationship of the L2 norm between target groups is not consistent with identification accuracy).

**Normal setting vs. Zero-shot setting** To confirm the performance limit in zero-shot neural decoding settings, we evaluate the performance of the proposed model in normal settings, where target groups are included in the training set (i.e., the original setting of dataset [6]). Table 6 shows the decoding performance of our model when each target group is included in the training set (normal) and not included in the training set (zero-shot). The results are averaged across five subjects. We can see that the performance in the normal setting is higher than in the zero-shot setting for all target groups. Although there is still a gap in performance between the normal and zero-shot settings, the proposed method achieves comparable performance for several target groups. (e.g., *bird*, *container*, and *equipment*). It is a natural result that the normal setting outperforms the zero-shot setting, and we found that the performance of the zero-shot setting is comparable to that of the normal setting for several target groups.

**Balance between visual and semantic features** We investigate the balance between visual and semantic features in decoding each target group. Table 7 and Table 8 show the best trade-off parameters η representing the importance of visual and semantic features in the normal and zero-shot settings, respectively. In the tables, visual features are important when the value is large, while semantic features are important when the value is small. From Table 7 in the normal setting, we see that the visual and semantic features are equally important in all target groups (i.e., η is around 0.5). On the other hand, Table 8 in the zero-shot setting shows that η is changed from the normal setting in some target groups (e.g., *mammal*, *invertebrate*, and *device*). In groups of *living thing* (i.e., *mammal*, *bird*, and *invertebrate*), η tends to become smaller, i.e., the importance of semantic features increases. In groups of *artifact* (i.e., *device*, *container*, *equipment*, *structure*, and *commodity*), η tends to become larger, i.e., the importance of visual features increases. The results suggest that visual features of *living thing* and semantic features of *artifact* suffer from the projection domain problem in the zero-shot setting. Therefore, we can see that another view (i.e., semantic features of *living thing* and visual features of *artifact*), which is less affected by the problem, contributes to the zero-shot neural decoding.

**Figure 5:**
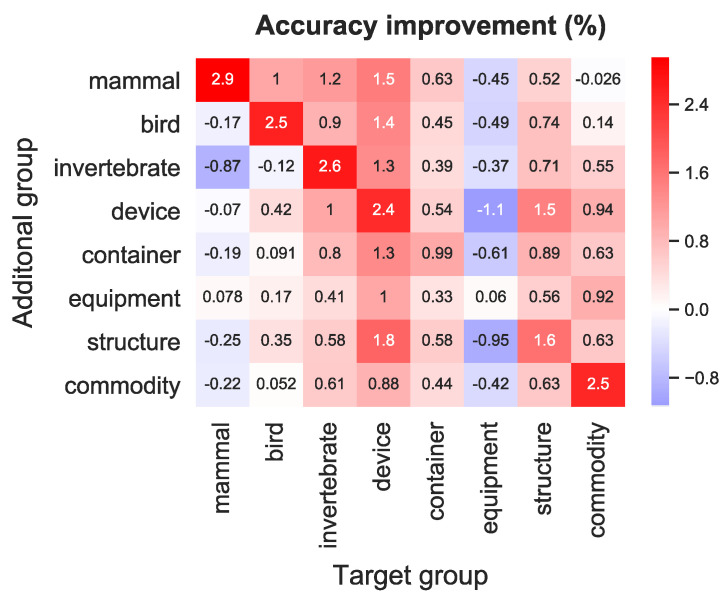
Identification accuracy improvement compared with MGM for all combinations of additional and target groups.

**Different additional group than the target group** In the above results, we utilized additional image categories belonging to each target group. Here, we discuss the decoding performance when incorporating a different additional group than the target group. Figure 5 shows the identification accuracy improvement of the proposed model for all combinations of additional and target groups in comparison with the accuracy of MGM. Accuracy improvement was averaged across five subjects. From the result, we confirmed that the method using the same additional group as the target group is the best model, while the accuracy is improved when the additional group is similar to the target group (e.g., *device* and *structure*). On the other hand, the utilization of other additional groups degrades the decoding performance when the target group is *equipment*. Thus, as expected, it is important to add categories that are relevant to the target group.

**Figure 6:**
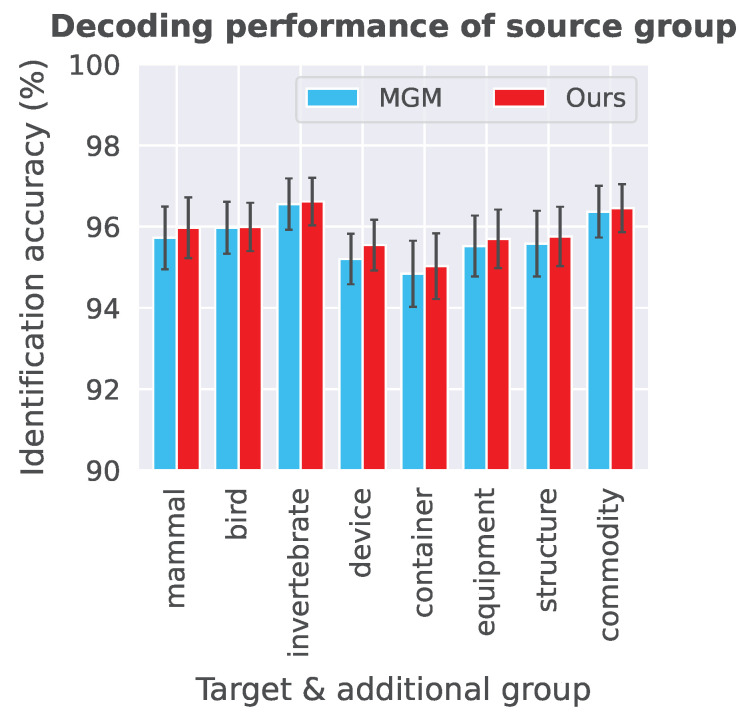
Decoding performance of the source group for each additional group in MGM and our proposed model. For example, when the target and additional groups are *mammal*, the source group is the other seven groups except for *mammal*. The identification accuracy was averaged across five subjects and the error bars represent standard errors.

**Effect of decoding performance on the source group** We investigate the decoding performance of the source group when each additional group is incorporated. Figure 6 shows the decoding performance of the source group for each target and additional group (the additional group is the same as the target group) in MGM and the proposed model. Note that the source group means the other seven groups except for each target and additional group. The result indicates that the utilization of additional image categories slightly improves the decoding performance of the source group. Therefore, these results give us confidence that the semi-supervised approach contributes to the decoding of the target group and does not negatively affect the decoding of the source group.

## 5. Conclusions

This paper has proposed a semi-supervised multi-view embedding approach for zero-shot neural decoding. In zero-shot neural decoding, the training data are scarce because of the difficulty in collecting brain activity patterns; therefore, the projection domain shift problem between the source domain and the target domain becomes remarkable. We address this problem by introducing the semi-supervised approach that employs additional image categories related to the target domain. Furthermore, to exploit the complementary information, we assume that fMRI activity patterns are projected into the visual feature space and semantic feature space. The experimental results show the advantages of the proposed model over existing methods.

Given the difficulty in collecting fMRI activity, we incorporate additional visual and semantic features from abundant images while estimating corresponding fMRI activity patterns. This framework can be applied to not only neural decoding tasks but also other difficult learning tasks where one type of modal data are insufficient whereas other modal data are available abundantly.

## Figures and Tables

**Table 1:** Meaning and role of observed variables and model parameters.

Symbol	Meaning	Role
Observed variables (k∈{f,v,s}):		
$X^{\left(f\right)}$	fMRI activity	Input for neural decoding
$X^{\left(v\right)}$	Visual features	Visual information of viewed image
$X^{\left(s\right)}$	Semantic features	Semantic information of viewed image
$X_{\mathrm{add}}^{\left(v\right)}$	Additional visual features	Alleviate the projection domain shift problem
$X_{\mathrm{add}}^{\left(s\right)}$	Additional semantic features	Alleviate the projection domain shift problem
${\hat{X}}^{\left(k\right)}$	Concatenated observed variables	Concatenate original and additional features
Model parameters (k∈{f,v,s}):		
Z	Shared latent variables	Link ${\hat{X}}^{\left(f\right)}$ with ${\hat{X}}^{\left(v\right)}$ and ${\hat{X}}^{\left(s\right)}$
$X_{\mathrm{miss}}^{\left(f\right)}$	Unobserved fMRI activity	fMRI activity corresponding to additional features
$W^{\left(k\right)}$	Projection matrix	Project Z into ${\hat{X}}^{\left(f\right)}$, ${\hat{X}}^{\left(v\right)}$, and ${\hat{X}}^{\left(s\right)}$
$\alpha^{\left(k\right)}$	Hyperprior distribution for $W^{\left(k\right)}$	Control the variance of $W^{\left(k\right)}$
$\beta_{k}$	Inverse variance for ${\hat{X}}^{\left(k\right)}$	Control the noise of ${\hat{X}}^{\left(k\right)}$

**Table 2:** Numbers of training, test, and additional categories for each target group.

	*Mammal*	*Bird*	*Invertebrate*	*Device*	*Container*	*Equipment*	*Structure*	*Commodity*
# Training	134	144	138	106	135	135	145	144
# Test	6	3	4	8	7	3	4	6
# Additional	1077	814	626	1038	639	417	1100	551

**Table 3:** Decoding performance in 10,000 random candidate categories. The first line for each method represents the identification accuracy (%) and the second line represents the standard error of five subjects. Existing neural decoding methods (SLR, CCA, Semi-FDCCA, and MLP), a part of the proposed model (BCCA-V, BCCA-S, and MGM), and the proposed model are separated by blocks.

Method	Target Group							
	*Mammal*	*Bird*	*Invertebrate*	*Device*	*Container*	*Equipment*	*Structure*	*Commodity*
SLR [6]	49.29 ± 4.17	83.90 ± 2.27	85.50 ± 2.26	87.11 ± 1.83	96.67 ± 0.33	94.25 ± 1.31	90.72 ± 1.08	87.39 ± 3.02
CCA [14]	76.90 ± 4.38	73.12 ± 2.43	74.85 ± 6.98	78.23 ± 2.11	87.20 ± 1.01	77.73 ± 1.89	72.44 ± 7.23	73.61 ± 5.86
Semi-FDCCA [7]	85.38 ± 0.91	86.60 ± 2.42	89.73 ± 0.98	83.76 ± 1.94	89.43 ± 2.42	91.95 ± 3.86	77.67 ± 6.45	80.10 ± 3.57
MLP [8]	75.14 ± 2.31	95.73 ± 0.98	91.25 ± 1.91	89.98 ± 1.65	97.63 ± 0.32	92.74 ± 1.34	90.90 ± 0.94	88.44 ± 1.98
BCCA-V [19]	45.65 ± 3.60	84.41 ± 3.68	82.90 ± 3.46	88.34 ± 1.78	96.56 ± 0.51	93.81 ± 0.79	89.13 ± 1.04	85.99 ± 2.49
BCCA-S [19]	83.51 ± 0.38	94.22 ± 0.48	84.64 ± 0.41	73.54 ± 1.71	65.39 ± 1.54	82.48 ± 1.32	77.14 ± 0.95	81.03 ± 0.46
MGM	83.97 ± 0.28	95.15 ± 0.59	90.46 ± 1.09	89.18 ± 1.50	97.19 ± 0.38	95.30 ± 0.61	91.47 ± 0.37	89.14 ± 1.62
**Ours**	**86.91** **± 0.33**	**97.69** **± 0.49**	**93.10** **± 1.20**	**91.61** **± 1.49**	**98.19** **± 0.23**	**95.36** **± 0.63**	**93.11** **± 0.22**	**91.63** **± 1.55**

**Table 4:** Rank-*n* accuracy (%) in 10,000 random candidate categories. The results are averaged across all target groups and five subjects.

Metric (Chance Level)	SLR	CCA	Semi-FDCCA	MLP	BCCA-V	BCCA-S	MGM	Ours
Rank-100 accuracy (1.0%)	16.03	9.092	11.58	20.76	14.32	0.4167	21.32	**30.22**
Rank-1000 accuracy (10%)	58.35	41.41	56.67	71.10	57.11	31.24	70.16	**77.66**
Rank-5000 accuracy (50%)	91.77	82.72	94.64	97.81	90.63	94.06	98.75	**99.06**

**Table 5:** Decoding performance in similar candidate categories. The first line for each method represents identification accuracy (%) and the second line represents the standard deviation of five subjects.

Method	Target Group							
	*Mammal*	*Bird*	*Invertebrate*	*Device*	*Container*	*Equipment*	*Structure*	*Commodity*
SLR [6]	65.18 ± 3.03	86.42 ± 4.86	75.17 ± 5.81	81.31 ± 3.00	92.75 ± 2.07	76.96 ± 9.11	79.96 ± 3.35	92.85 ± 2.86
CCA [14]	60.29 ± 1.77	73.62 ± 16.38	65.88 ± 17.45	75.17 ± 6.50	82.93 ± 3.29	71.35 ± 4.48	59.99 ± 16.58	69.38 ± 13.15
Semi-FDCCA [7]	73.34 ± 3.30	80.50 ± 9.93	79.18 ± 5.45	79.44 ± 6.65	82.87 ± 7.17	80.90 ± 12.12	60.40 ± 11.25	83.01 ± 7.30
MLP [8]	73.98 ± 3.62	91.26 ± 2.34	80.42 ± 6.76	86.57 ± 3.28	94.23 ± 1.93	75.40 ± 7.28	76.61 ± 4.44	92.59 ± 2.30
BCCA-V [19]	65.38 ± 1.61	83.90 ± 2.77	74.06 ± 6.06	84.41 ± 5.20	92.80 ± 2.53	73.99 ± 5.89	77.80 ± 2.41	93.42 ± 1.49
BCCA-S [19]	61.83 ± 1.51	78.79 ± 2.70	58.96 ± 2.90	74.80 ± 1.94	50.63 ± 4.11	86.23 ± 0.96	87.14 ± 1.00	87.04 ± 0.27
MGM	74.68 ± 2.10	92.62 ± 2.80	78.71 ± 5.08	87.11 ± 3.31	93.48 ± 1.94	90.14 ± 1.56	88.59 ± 1.13	95.87 ± 0.68
**Ours**	**77.38** **± 2.72**	**94.45** **± 1.86**	**82.54** **± 6.14**	**90.46** **± 2.59**	**95.07** **± 1.33**	**90.37** **± 1.58**	**88.62** **± 1.98**	**96.03** **± 0.77**

**Table 6:** Identification accuracy (%) of the proposed model for normal and zero-shot settings.

	*Mammal*	*Bird*	*Invertebrate*	*Device*	*Container*	*Equipment*	*Structure*	*Commodity*
Normal	96.49	98.84	97.28	95.33	98.88	97.58	97.04	94.21
Zero-shot	86.91	97.69	93.10	91.61	98.19	95.36	93.11	91.63

**Table 7:** The trade-off parameter η of the proposed model in the normal setting. Visual features are important when the value is large, while semantic features are important when the value is small.

	*Mammal*	*Bird*	*Invertebrate*	*Device*	*Container*	*Equipment*	*Structure*	*Commodity*
Subject 1	0.3	0.5	0.4	0.7	0.8	0.4	0.5	0.3
Subject 2	0.7	0.8	0.7	0.6	0.7	0.5	0.5	0.5
Subject 3	0.7	0.7	0.8	0.6	0.8	0.4	0.5	0.6
Subject 4	0.5	0.7	0.7	0.5	0.8	0.5	0.6	0.7
Subject 5	0.6	0.7	0.7	0.6	0.8	0.5	0.6	0.5
Mean	0.56	0.68	0.66	0.60	0.78	0.46	0.54	0.52

**Table 8:** The trade-off parameter η of the proposed model in the zero-shot setting. Visual features are important when the value is large, while semantic features are important when the value is small.

	*Mammal*	*Bird*	*Invertebrate*	*Device*	*Container*	*Equipment*	*Structure*	*Commodity*
Subject 1	0	0.4	0.3	0.7	0.8	0.4	0.5	0.3
Subject 2	0	0.7	0.6	1.0	0.7	0.7	0.6	0.5
Subject 3	0.3	0.8	0.6	0.7	0.7	0.4	0.5	0.7
Subject 4	0.2	0.7	0.5	0.6	0.9	0.5	0.7	0.7
Subject 5	0	0.3	0.4	0.7	0.8	0.5	0.6	0.6
Mean	0.10	0.58	0.48	0.74	0.78	0.5	0.58	0.56

## Data Availability

The public dataset used in our experiment is available at https://github.com/KamitaniLab/GenericObjectDecoding (accessed on 15 April 2023).
